# *Bacillus megaterium* GXU087 secretes indole - 3 - lactic acid to promote soybean growth and nodulation

**DOI:** 10.3389/fpls.2025.1560346

**Published:** 2025-03-21

**Authors:** Jingsi Qiu, Xiao’ou Meng, Jingdong Li, Tengfei Zhang, Siying Qin, Yuanfu Li, Huihua Tan

**Affiliations:** Guangxi Key Laboratory for Agro-Environment and Agric-Product Safety, National Demonstration Center for Experimental Plant Science Education, College of Agriculture, Guangxi University, Nanning, Guangxi, China

**Keywords:** soybean, *Bacillus megaterium*, indole-3-lactic acid, PGPB, nodulation

## Abstract

*Bacillus* species are recognized as plant growth-promoting bacteria (PGPB), yet the mechanisms behind their crop growth promotion remain elusive. This study is designed to explore the plant growth-promoting (PGP) effects of *Bacillus megaterium* GXU087 on soybeans and to uncover the underlying mechanism. *In vitro*, GXU087 exhibited various PGP traits, including phosphate solubilization, nitrogen fixation, production of exopolysaccharide, and biofilm formation. Pot experiments indicated that GXU087 significantly enhanced soybean growth, leading to a remarkable increase in fresh weight (*p* < 0.05). Additionally, the nodulation parameters of soybeans were improved. Specifically, a 10% concentration of the extracellular extracts from GXU087 exerted a significant promotion on nodulation, with both nodule number and fresh weight parameters increasing significantly (*p* < 0.05). UPLC-MS analysis verified that GXU087 secreted indole-3-lactic acid (ILA) at a concentration of 232.7 ng/mL. Pot assays further demonstrated that this ILA effectively promoted soybean growth and nodulation within a concentration range of 0.1-10 mg/L. However, exogenous application of ILA did not stimulate rhizobia reproduction, suggesting that bacterial ILA does not promote nodulation by enhancing rhizobia growth. Notably, this is the first report of *B. megaterium* secreting ILA as a growth and nodulation promoter in soybeans. Our findings offer new insights into the mechanism of *B. megaterium* action and contribute to the understanding of microbe-induced growth promotion in crops.

## 1 Introduction

Soybean (*Glycine max*) is a crucial *Leguminosae* crop, serving as a major source of plant oil for human consumption (Liu et al., 2020; Xu et al., 2020). Various rhizobacteria like *Rhizobium*, *Bradyrhizobium*, and *Azotobacter* have been widely used as bio-inoculants to promote *Leguminosae* growth and development (Luisa et al., 2024). These Plant Growth Promoting Rhizobacteria (PGPRs) enhance crop yield and tolerance through improving nutrient absorption, degrading organic pollutants, and producing phytohormones, antibiotics, vitamins, and siderophores (Numan et al., 2018). Plant Growth Promoting Bacteria (PGPBs) encompass a broader category of bacteria that play a crucial role in facilitating plant growth. Genera such as *Pseudomonas*, *Enterobacter*, and *Bacillus*, which are part of PGPB group, have been extensively researched and applied in both normal and stressful conditions (Peng et al., 2023; Fanai et al., 2024). These beneficial PGPB can produce various phytohormone like indole-3-acetic acid (IAA), gibberellic acids, cytokinin (CK) and abscisic acid, as well as ammonia, amino acids, exopolysaccharides (EPS), and 1-aminocyclopropane-1-carboxylate (ACC) deaminase to promote plant growth (Pankievicz et al., 2021). Various reports have demonstrated that PGPB promote crop growth by increasing protein accumulation and facilitating the uptake of nitrogen, potassium, and phosphorus (Solouki et al., 2023; Zheng et al., 2019; Rawat et al., 2020), and producing siderophores and antibiotics to protect plants from pathogens under stressful conditions (Timofeeva et al., 2022). Moreover, many PGPBs modulate the gene expression and the metabolism of stress-protective agents to enhance plant stress tolerance (Qin et al., 2018; Sharma et al., 2019).

*Bacillus* sp. is commonly used as PGPBs in agricultural fields due to their excellent traits in survival and plant growth promotion (Hashem et al., 2019; Xiong et al., 2020; Kazerooni et al., 2021). For instance, a particular strain of *Bacillus* sp. has been found to produce IAA and salicylic acid (SA), leading to significant growth promotion in maize and wheat under drought stress (Jochum et al., 2019). The combined application of two *Bacillus* strains has been shown to enhance the resilience of drought-stressed rice by quenching the reactive oxygen species (Narayanasamy et al., 2020). *Bacillus* sp. has been demonstrated to be beneficial in association with soybean crops, with studies revealing that this genus positively influences root nodulation and *Leguminosae* growth (Tilak et al., 2006; Bai et al., 2002). For example, the combined use of rhizobacteria *Bacillus* with *Rhizobium* has been shown to have a synergistic effect on the growth, disease control and mitigate Nickel toxicity in the common bean and Faba Bean plants (Ferreira et al., 2020; Korir et al., 2017; Elbagory et al., 2022). Inoculation of *B. cereus* GS6 in combination with phosphate-enriched compost have significantly increased the nutrient uptake in soybean plants, and improved dehydrogenase and phosphomonoesterase activities, as well as the available N, P, and K contents in soil (Arif et al., 2017). Although the positive influences of *Bacillus* on leguminous growth and nitrogen fixation have been extensively studied, there is still limited information available on how those *Bacillus* strains promote the soybean growth and nodulation (Kumar et al., 2021; Miljaković et al., 2022a; Sahile et al., 2021). Sibponkrung et al. (2020) found that the disruption of genes involved in IAA and CK biosynthesis in *B. velezenis* strain S141 led to smaller nodules in soybean-*Bradyrhizobium* symbiosis, proving the important role of hormones secreted by S141 in the nodule growth. Nevertheless, our comprehension of the molecular mechanisms underlying the interaction between *Bacillus* species and plants is profoundly restricted. The elucidation of additional mechanisms contributing to the phytostimulation induced by *Bacillus* remains an area of considerable interest.

In this study, we isolated and identified a novel PGPB strain, *B. megaterium* GXU087, which exhibit various plant growth-promoting (PGP) traits. This strain promotes the growth and nodulation of soybean by secreting extracellular metabolites, specifically identified as ILA (indole-3-lactic acid). Importantly, this is the first reported of *B. megaterium* promoting nodulation in soybean by producing ILA, and our findings provide novel insight into the PGP mechanism of *B. megaterium*.

## 2 Materials and methods

### 2.1 Chemicals

The DF medium was purchased from Beijing Coolaber (Beijing, China), glutaraldehyde from Kelong Chemical (Chengdu, China), IAA standard from ABPHYTO biotechnology (Chengdu, China) and CAS medium from Hope Bio-Technology (Qingdao, China). The Yeast Mannitol medium (YMM) consist of mannitol (10 g/L), MgSO_4_·7H_2_O (0.2 g/L), NaCI (0.1 g/L), K_2_HPO_4_·3H_2_O (0.5 g/L), yeast extract (0.8 g/L) with the pH adjusted to 6.9-7.2. The Luria-Bertani (LB) agar media comprise tryptone (10 g), yeast extract (5 g), NaCl (10 g), and agar (15 g) in 1 L of distilled water, with the pH adjusted to 7.0. The Mongina medium is formulated with dextrose (10 g/L), (NH4)_2_SO_4_ (0.5 g/L), NaCl (0.3 g/L), KCl (0.3 g/L), FeSO_4_·7H_2_O (0.03 g/L), MgSO_4_·4H_2_O (0.03 g/L), CaCO_3_ (5 g/L), yeast extract (0.4 g/L), egg yolk lecithin (0.2 g/L), and agar (15 g/L), with the pH adjusted to 7.

### 2.2 Screening for plant-growth bacteria and phenotypic characterization of candidate isolates

Soil samples were collected from a field at the Dry Grain Crop Research Institute, Guangxi Academy of Agricultural Sciences (Guangxi, China), where soybean plants have been cultivated for several years. The freshly collected soil was stored at 4°C, and solid debris was later removed in the laboratory. Five grams of treated soil were mixed with sterilized water in a tube, vigorously shaken, and incubated at 85°C for 10 minutes. Subsequently, the broth was diluted and plated on LB agar media. Various morphological colonies were selected and purified through repeated sub-culturing in LB media. The obtained purified strains were preserved and assayed for plant growth-promoting capabilities. Ultimately, a strain exhibiting the highest plant-promoting ability was identified and designated as GXU087. GXU087 was characterized using a stereomicroscope and scanning electron microscopy (SEM). This strain was cultured on LB plates and incubated at 28°C for two days for bacterial-colony observation. SEM imaging was conducted to analyze the microstructural characteristics of GXU087. Bacterial samples were prepared by fixing them with 2.5% glutaraldehyde and incubating at 4°C for 2 hours, followed by dehydration using a series of ethyl alcohol solutions (30%, 50%, 70%, 90%, and 100% of ethyl alcohol) and finally freeze-dried. The samples were then mounted and sputter-coated with gold before imaging with a field emission scanning electron microscope (Quattro S, Thermo Fisher) at 10,000×magnifications and 7 kV.

### 2.3 The growth curve and genotypic characterization of GXU087 isolates

The growth curve of bacteria was assessed using a BIOSCREEN C° PRO system (Oy Growth Curves Ab Ltd., Turku) with germ-free 96-well microtiter plates. Bacterial strains were cultured in LB medium for 24 hours. Bacterial pellets were harvested by centrifugation and resuspended in sterilized deionized water (SDW) to achieve a bacteria solution with an OD_600_ of 0.1. Each well was loaded with 10 μL of the bacterial solution and 190 μL of fresh LB medium. The 96-well microtiter plates were secured on the sample tray and incubated at 28°C with orbital shaking for 24 hours. The OD_600_ value of each well was recorded every 60 min, and the growth curve was plotted with time on the x-axis and OD_600_ value on the y-axis. The experiment was repeated three times with five replicates each. In this study, the 16S rRNA gene and whole genome sequence of the GXU087 strain was identified. High-quality genome extraction was performed using a NucleoBond^®^ HMW DNA kit (MN NucleoBond, Germany). The DNA concentration, purity and integrity were determined using a Qubit4.0 (Thermo Fisher Scientific, Q33226), Nanodrop (SMA4000, Taiwan, China), and 0.75% agarose gel electrophoresis, respectively. The genome sequence of the candidate strain was obtained through Single-molecule Nanopore DNA sequencing (MinION Flow Cell (ONT, R9.4.1)) and the MGI DNBSEQ-T7 platforms by Shenggong Co., Ltd. (Shanghai, China). Coding sequences (CDSs) in the strains were predicted using Prokka (Version 1.10) and functionally annotated with the NCBI database.

### 2.4 PGP traits of GXU087 isolates

To assess the PGP traits of the GXU087 strain, the strain was cultured in LB medium until it reached the logarithmic growth phase. A concentration of 10^6 colony-forming units (CFU) per milliliter was used for the PGP traits analysis.

For the assessment of inorganic phosphate solubilization, Pikovskaya’s agar plates were used (Amna et al., 2020). For organophosphate solubilization, Mongina medium plates were used. Bacterial cultures were spot-inoculated onto the plate centers and incubated for several days. The formation of a halo zone around the inoculation spot indicated that the strain could solubilize phosphate. Chrome azurol S (CAS) agar media was used to assess siderophore production (Ali et al., 2022). The bacterial culture was spot-inoculated on the CAS plate, and siderophores production was identified by the emergence of orange zones around the bacterial colonies.

Ammonia production was monitored following the method described by Amna et al. (2020). Potassium solubilization and ACC deaminase enzyme activity were assayed as outlined by Kumar et al. (2012). Exopolysaccharide production was evaluated using ATCC medium No.14 as described by Amna et al. (2019). The bacterial culture was spread on ATCC medium No.14 and incubated for several days. The presence of slime produced by colonies was regarded as positive for EPS production.

Nitrogen fixation was assessed following Timofeeva et al. (2023), with positive results indicated by strong growth and halo zone on Ashby’s Mannitol plates.

Biofilm formation on plastic surfaces was conducted as described by Ali et al. (2022) with some modifications. The GXU087 strains were grown in LB broth for 24 hours, then diluted to an OD_600_ of 1 in a phosphate-buffered saline (PBS)solution. Polyvinylchloride 96-well microtiter dishes were used for biofilm development, with each well filled with 100 μL of bacterial culture solution and 100 μL of Msgg medium (Li et al., 2018). The negative control group was treated with sterile Msgg medium without bacteria. Bacteria was incubated at 28 °C with constant humidity for 18 hours, and growth was measured using OD_600_ value. The microstructure of the bacterial biofilm was observed using an inverted microscope (xiovert 5, Zeiss). All of the above indicators were tested with three biological replicates and three technical replicates. This assay was repeated two or three times to ensure the reliability of positive results.

### 2.5 Plant growth and nodulation promotion assays

#### 2.5.1 Pot experiment on soybean growth and nodulation with rhizobia and GXU087 strain Inoculations

*Bradyrhizobium japonica* USDA110 strain (hereinafter referred to as rhizobia) and GXU087 strains were utilized in the pot experiment. Inoculants were prepared by culturing rhizobia in YMM medium and GXU087 in LB medium at 28°C with agitation until an OD_600_ of 1 was achieved. For the co-inoculation treatment, rhizobia and GXU087 inoculants were mixed in a 1:1 ratio.

Soybean seeds (*Glycine max* var. *zhonghuang 13*) were sterilized in 5% ethyl alcohol for 1 minute and 95% sodium hypochlorite for 10 minutes, followed by five rinses with SDW to remove any residual sanitizer. The sterilized seeds were then germinated in a mixture of autoclaved vermiculite and commercial nursery soil (1:1 volume ratio) in a nursery plate and cultivated in a greenhouse for approximately 7 days. The seedings were watered daily with sterile water and grown under a 16-hour photoperiod after germination. After 7 days, seedlings with two euphylla were carefully selected and transplanted into pots containing the mixture of autoclaved vermiculite and nursery soil. All seedlings were grown under a 16-hour light and 8-hour dark cycle, with temperatures maintained at 26°C during the day and 20°C at night. The relative humidity of the soil was kept at 70% ± 2% until harvest. Three days after transplanting, each pot was inoculated with 30 mL of either a single strain culture or a mixed strains culture (10^6 CFU/mL) for the initial inoculation. The treatments included: inoculation without bacteria (CK); mono-inoculation with rhizobia inoculation (R); mono-inoculation with GXU087 strain (GXU087); and co-inoculation with rhizobia and GXU087 strain (R+ GXU087). A week later, a second inoculation of 30 mL of fresh bacterial culture was administered. Plants without bacteria inoculation served as the control group. After 14 days post-bacterial inoculation (dpi), plants were harvested to assess growth parameters, including (a) whole plant fresh weight; (b) shoot fresh weight; (c) root fresh weight. At 21 dpi, plants were harvested to assess the nodulation parameters, including, (d) nodules number; and (e) nodules fresh weight. The experiment was conducted in triplicate with six replicates for each treatment. Significance analysis was performed using ANOVA, followed by the Turkey-Kramer Test for all pairs.

#### 2.5.2 Investigation of the effects of extracellular extracts from GXU087 bacteria on the growth and nodulation of soybean plants

Soybean seedlings were grown and selected as described in Section 2.5.1. The GXU087 stain was inoculated into LB medium and cultured for 48 hours. Subsequently, extracellular extracts were obtained after the removal of bacterial cells. Gradient concentrations of these extracellular extracts were tested in a preliminary experiment. In this study, 10% and 50% concentration of the extracellular extracts (designated as 10% E and 50% E, respectively) were utilized. The effect of the extracellular extracts on the growth and nodulation of soybean plants was investigated. Specifically, 30 mL of germ-free extracellular extracts broth was irrigated into the roots of seedlings. The control group received irrigation with SDW and LB medium. Growth and nodulation parameters were examined as detailed in Section 2.5.2. The repetitions and statistical analysis conducted were consistent with those outlined in Section 2.5.2.

#### 2.5.3 Analysis of extracellular extracts of GXU087 bacteria Using UPLC-MS

To investigate potential stimulants that significantly promote plant growth and nodulation, we analyzed the extracellular extracts of GXU087 using Ultra high-performance liquid chromatography-mass spectroscopy (UPLC-MS). Extracellular exudates were analyzed using an ultra-high-performance liquid chromatography system (ExionLC™ AD) coupled with a tandem triple quadrupole mass spectrometer (QTRAP^®^ 6500+). Separation was achieved on a Waters ACQUITY UPLC HSS T3 C18 column (1.8 μm, 100 mm×2.1 mm i.d.) with a column temperature set at 40°C and a flow rate of 0.35 mL/min. The injection volume was 2 μL. The mobile phase for the gradient elution consisted of A: water (containing 0.04% v/v acetic acid) and B: methanol (containing 0.04% v/v acetic acid) under a gradient flow. The gradient elution program was as follows: 0-1 min, 95% A; 1-8 min, linear gradient from 95%–5% A, held for 1 min; 9.1-12 min, linear gradient returning to the initial composition 95% A. The total run time was 12 min. Standard solution with different concentrations (0.01 ng/mL, 0.05 ng/mL, 0.1 ng/mL, 0.5 ng/mL, 1 ng/mL, 5 ng/mL, 10 ng/mL, 50 ng/mL, 100 ng/mL, 200 ng/mL, 500 ng/mL) was prepared. The standard curves of each substance are plotted with the concentration ratio between the external standard (the standard of each auxin to be measured) and the internal standard as the horizontal coordinate and peak area ratio as the vertical coordinate.

#### 2.5.4 Study on the physiological effects of Indole-3-lactic acid in soybean seedings

ILA, an indole derivative, serves as an auxin analogue of IAA due to its structural

similarity to the IAA. Abundant quantities of ILA were detected in the extracellular extracts, promoting an investigation into its potential PGP effects. In this study, a gradient of physiological concentrations ranging from 0.1 mg/L to 10 mg/L were utilized. Soybean seedlings were grown according to the methods described in Section 2.5.1. To compared the effects of ILA, 0.1 mg/L of IAA standard solution was used as positive control for auxin activity. The control groups were inoculated with SDW for growth assay for growth and rhizobia for nodulation assay. Growth and nodulation parameters were examined as detailed in Section 2.5.2. The experiment was conducted with six to ten replicates per treatment, and statistical significance analysis was analyzed using ANOVA followed by the Duncan Test for all pairwise comparisons.

#### 2.5.5 Effects of exogenous ILA on rhizobia growth

To investigate whether exogeneous ILA promotes the reproduction of rhizobia strain and subsequently enhances nodulation of soybean, an experiment was conducted to assess the impact of ILA on rhizobia growth utilizing a BioTek Epoch 2 system (Agilent, USA) and sterile 96-well microtiter plates. Rhizobia strains were cultivated in YMA medium for 48 hours. Following incubation, bacterial pellets were harvested to prepare a bacterial solution with an OD_600_ of 1.0. Each well of microtiter plates contained 200 μL of solution, comprising 50 μL of bacterial solution, YMA medium, and exogenous substances (IAA standard solution was used as positive controls) at concentrations ranging from 0.1 to 10 mg/L. The plates were secured on the sample tray and incubated at 28°C with orbital shaking for 48 hours. The OD_600_ value of each well was recorded at 0,6,12,24,36,48 hours. Growth curve was plotted as described in Section 2.3. The experiment was replicated three times with ten replicates per treatment.

## 3 Result

### 3.1 Phenotypic characterization and growth curve of GXU087 strain

**Figure 1:**
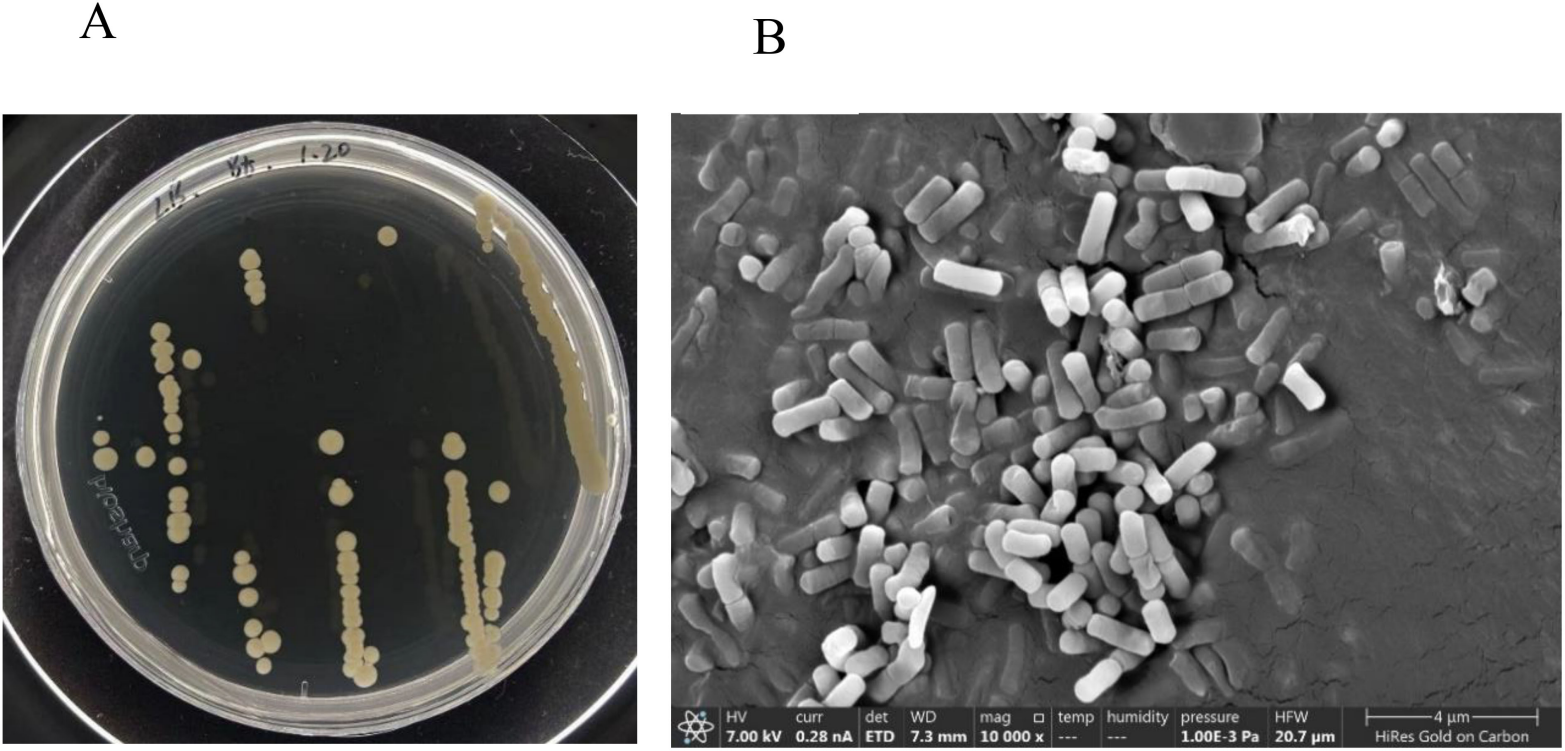
Colony morphology of GXU087 strain on the LB agar **(A)** plate and under SEM **(B)**.

From soil samples, several strains with plant growth-promoting traits were isolated, with the GXU087 strain exhibiting the highest plant-promoting ability. When grown on LB medium at 28 °C for 2 days, colonies of GXU087 appeared smooth and circular, with smooth borders and a color range from white to pale yellow (**Figure 1A**). SEM observations showed that the cells were rod-shaped, measuring approximately 1.3-1.8 μm in length and 0.5-0.6 μm in width of (**Figure 1B**). The growth dynamics of the bacteria are illustrated in **Supplementary Figure S1**. Initially, during the early phase (0-5h), the cells grew slowly. However, between 5-10 h, they entered a rapid growth phase during the logarithmic proliferation stage. From 10-24 h, the growth reached a stable stage, peaking at an OD_600_ of 1.4, with the reproductive and death rates remaining relatively stable throughout this period.

### 3.2 Phylogenetic analyses of GXU087 related to *Bacillus megaterium*

We constructed the Neighbor-Joining phylogenetic tree of GXU087 based on the 16S rRNA sequence, combined with similar homologous sequences retrieved on NCBI. The results, which are depicted in **Supplementary Figure S2**, indicate that GXU087 is most closely related to *Bacillus megaterium*. The whole genome sequence of the GXU087 strain is 5,212,177 bp in length and has a GC content of 38%. The genome of the GXU087 strain comprises a chromosome (5,140,930 bp) and two plasmids (6,182,5 bp and 9,422 bp) (see **Supplementary Figure S3**-**Supplementary Figure S5**). The genome sequences of the GXU087 strain have been deposited in NCBI under accession numbers CP144863-CP144865.

### 3.3 GXU087: a multifaceted PGPB with various beneficial traits

GXU087 is a biochemically characterized PGPB (Plant Growth-Promoting Bacterium) exhibiting various traits that promote plant growth, as detailed in **Table 1**. It demonstrated a remarkable ability to solubilize phosphate, evidenced by clear zone forming around colonies on Pikovskaya’s agar medium and Organic Phosphorus Fluid agar medium (see **Supplementary Figures S6A, B**). The ATCC medium revealed slime formation around colonies, suggesting exopolysaccharides production by GXU087 (**Supplementary Figure S6C**). Additionally, clear zones on Ashby plates indicate its nitrogen fixation capability (**Supplementary Figure S6D**). GXU087 also exhibited strong biofilm formation, with a uniform distribution of bacteria on plate surfaces and scattered cell clusters (**Supplementary Figure S7**). However, it did not produce siderophore or ammonia, as confirmed by negative results on CAS plates and Nessler’s reagent respectively (data not shown). Additionally, it did not dissolve potassium, as shown by negative Aleksandrov agar plates results (data not shown). This strain could not grow in minimal media with ACC as the sole nitrogen source, indicating no ACC deaminase production (data not shown). Overall, GXU087 is an exceptional PGPB with multiple beneficial traits for plant growth.

**Table 1:** Morphological, biochemical characteristics of strain *B. megaterium* GXU087 strain.

Characteristics studied	Results
Morphological/Colony characteristics	Large-sized, regular, subcircular, flat, off-white, smooth colonies on LB agar at 28°C after 24 h of incubation
Bacterial cell features	The gram-positive, microscopic view showed cells have a short rod shape
Biochemical properties	Positivefor phosphate solubilization (organophosphorus and inorganic phosphate), exopolysaccharide production, nitrogen fixation, and biofilm formation. Negative for siderophore production, ammonia production, potassium solubilization, and ACC deaminase activity.

### 3.4 GXU087 strain enhances soybean growth and nodulation, with synergistic effects when combined with rhizobia

**Figure 2:**
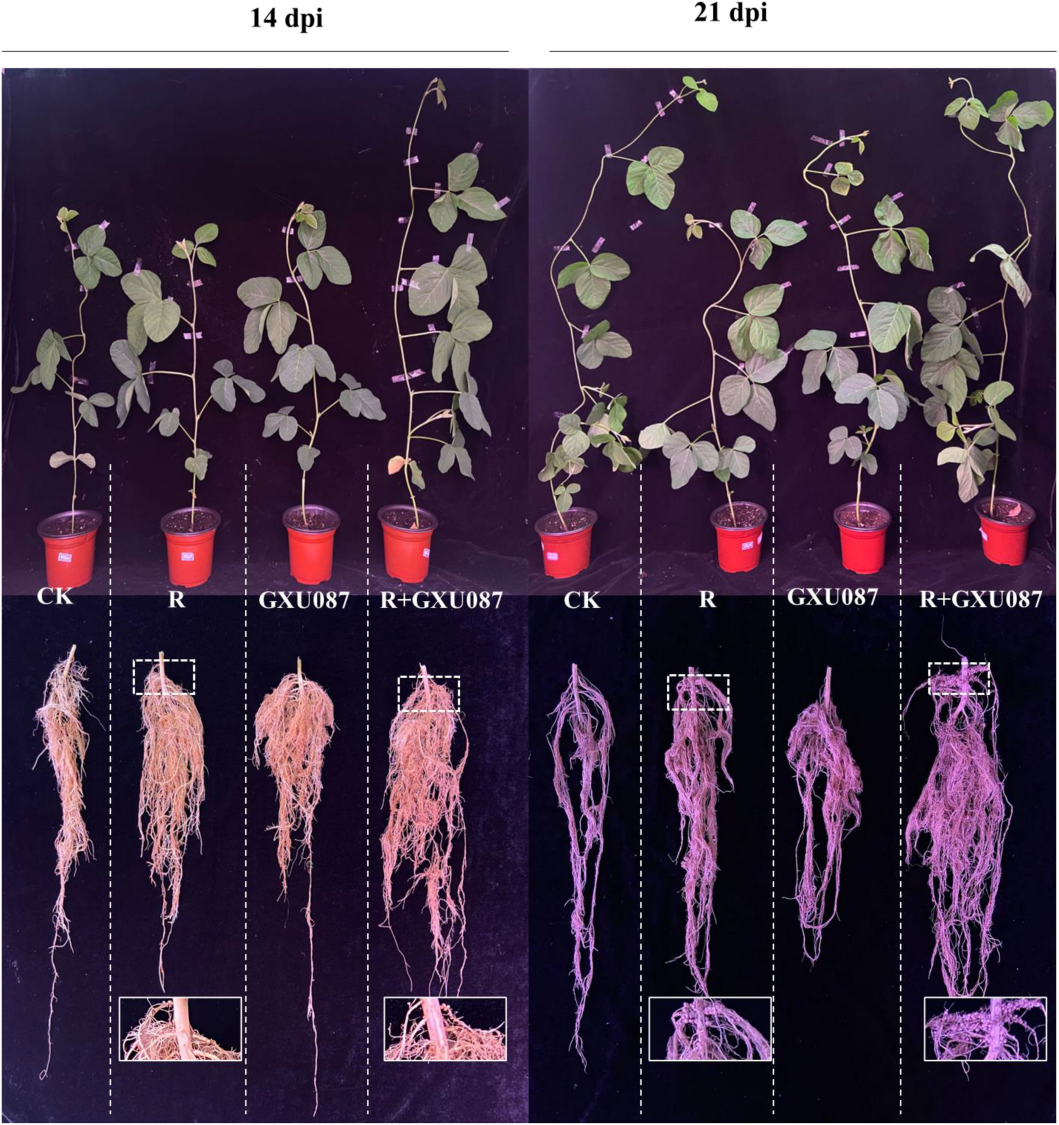
Overview of the growth promotion by GXU087 strain in soybean. The treatments included: inoculation without bacteria (CK); mono-inoculation with rhizobia inoculation (R); mono-inoculation with GXU087 strain (GXU087); and co-inoculation with rhizobia and GXU087 strain (R+ GXU087). n=6.

**Figure 3:**
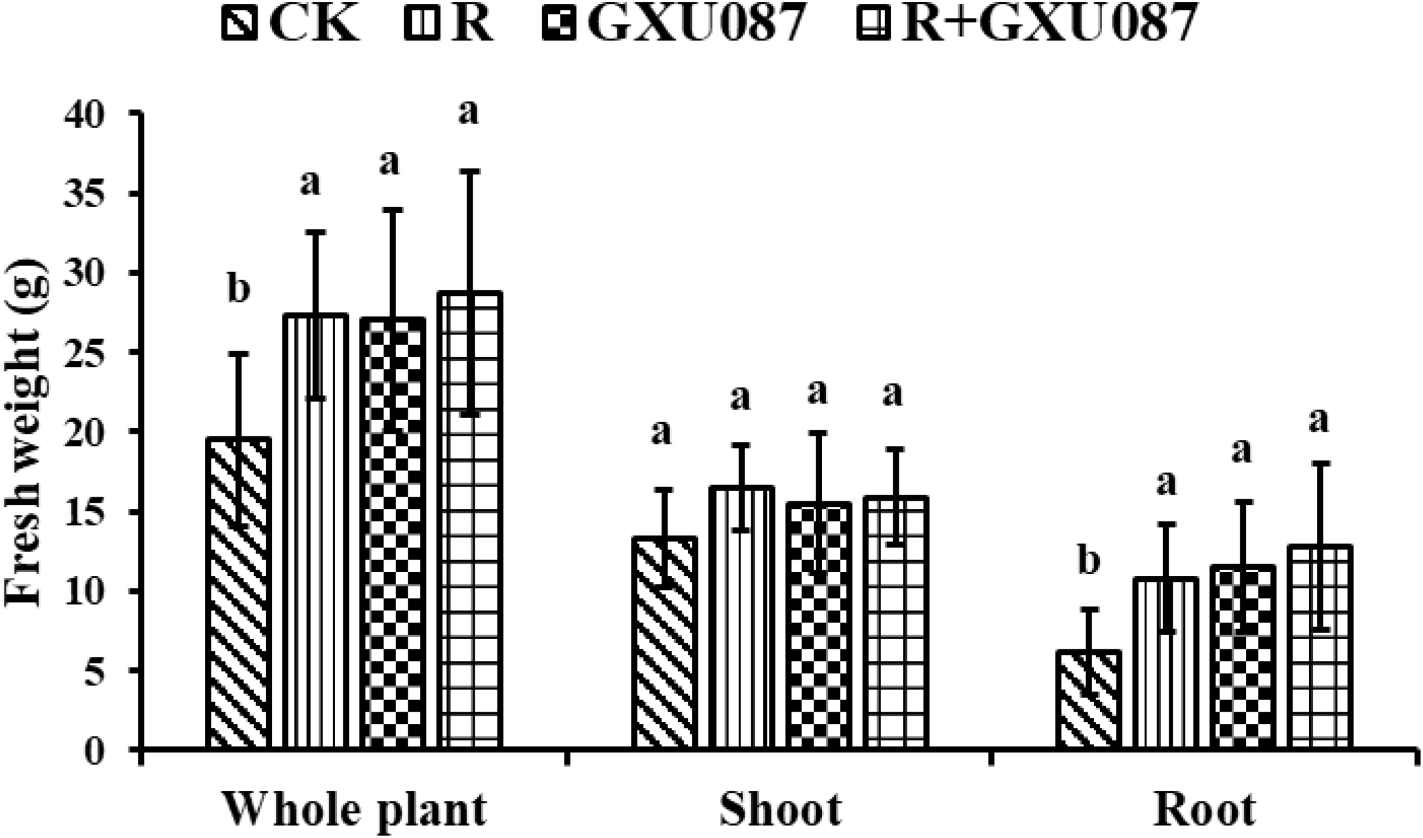
Growth promotion on the weight of soybean by GXU087 strain at 14 dpi. Significance at *p ≤0.05* is indicated by mean standard error bars (n=6). The treatments included: inoculation without bacteria (CK); mono-inoculation with rhizobia inoculation (R); mono-inoculation with GXU087 strain (GXU087); and co-inoculation with rhizobia and GXU087 strain (R+ GXU087). Lowercase letters in the figure show significant differences. Same letters mean non - significant group differences; different ones mean significant ones.

**Figure 4:**
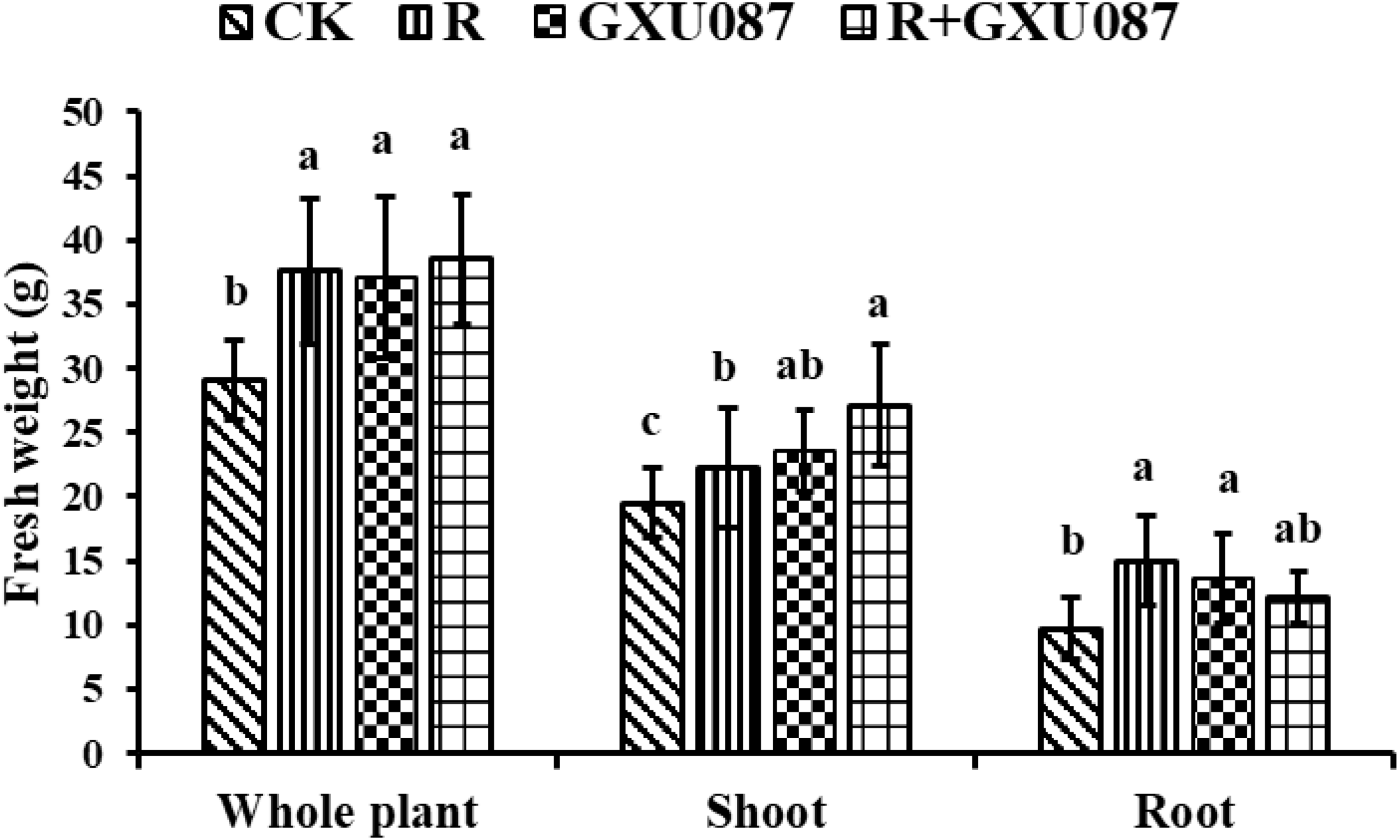
Growth promotion on the weight of soybean by GXU087 strain at 21 dpi. Significance at *p ≤0.05* is indicated by mean standard error bars (n=6). The treatments included: inoculation without bacteria (CK); mono-inoculation with rhizobia inoculation (R); mono-inoculation with GXU087 strain (GXU087); and co-inoculation with rhizobia and GXU087 strain (R+ GXU087). Lowercase letters in the figure show significant differences. Same letters mean non - significant group differences; different ones mean significant ones.

The impact of the GXU087 strain on soybean growth was evaluated by measuring plant weight and nodulation. **Figure 2** shows that soybean plants inoculated with the GXU087 strain exhibited greater growth in shoots and roots compared to control groups at 14 dpi and 21 dpi. The promotion effect of GXU087 is similar to that of rhizobia, a widely used PGPB for soybean. This demonstrates that GXU087 strain is also a PGPB for soybean plants. When combined with rhizobia, the two strains showed a synergistic effect on soybean growth, with more developed roots and larger leaves in the “R+GXU087” treatment compared to the control. As shown in **Figures 3**, **4**, the whole plant fresh weight and root fresh weight of GXU087-inoculated plants were significantly different from those of control plants. Specifically, the fresh weight of roots in the GXU087-inoculated and co-inoculated treatment at 14 dpi was 11.5 g and 12.8 g, respectively, compared to 6.2 g in control. This represented a significant increase of over 85% (*p* < 0.05). While the shoot fresh weight was higher in both treatments (15.5 g and 15.9g) versus the control (13.3 g), the difference was not statistically significant. Therefore, GXU087 primarily improves root biomass in soybean plants at the early stage.

At 21 dpi, shoots in the GXU087-inoculated and co-inoculated group weighted 27.1 g and 23.6 g, respectively, significantly heavier than the control group’s 19.5 g (*p* < 0.05). This suggests that GXU087 mainly promotes shoot growth in the later stage (**Figure 4**).

**Figure 5:**
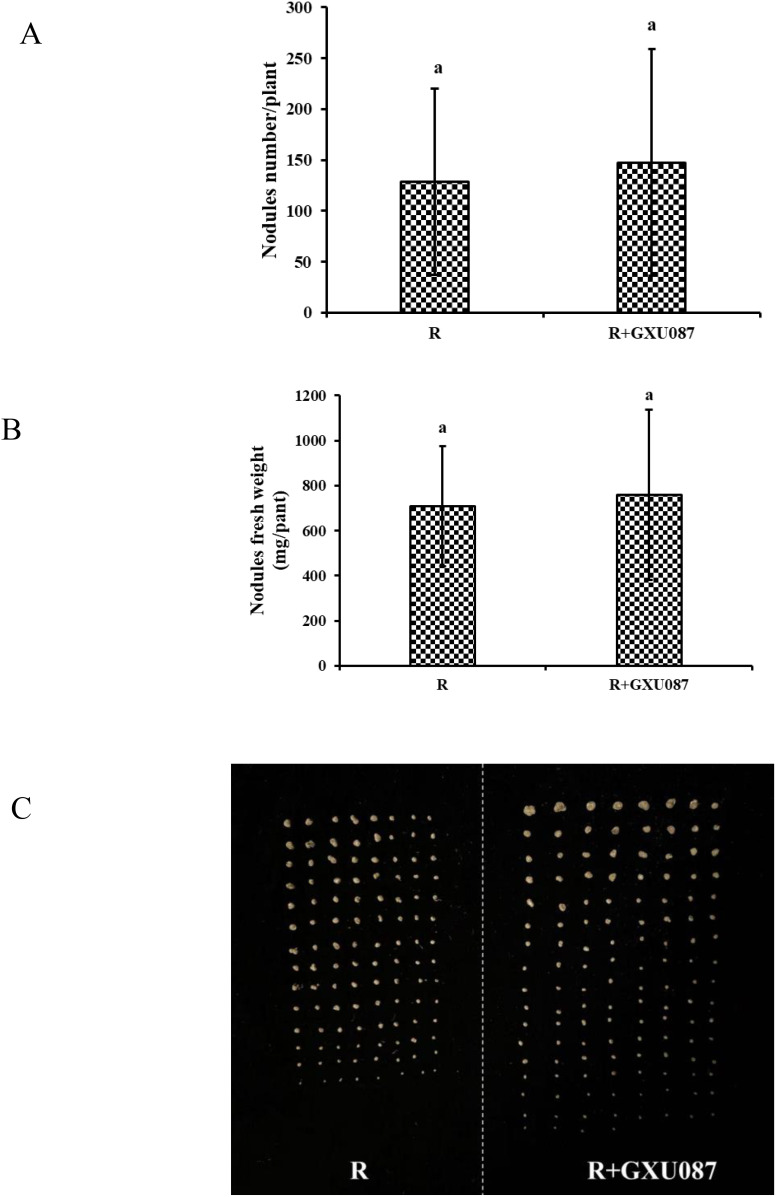
Nodulation parameter of soybean by co-inoculation with GXU087 and rhizobia at 21 dpi: **(A)** nodules number/plant; **(B)** nodules fresh weight; **(C)** photograph of soybean nodule. Significance at *p ≤0.05* is indicated by mean standard error bars (n=6). The treatments included: mono-inoculation with rhizobia inoculation (R) and co-inoculation with rhizobia and GXU087 strain (R+ GXU087). Treatments without bacteria (CK) and mono-inoculation with GXU087 alone (GXU087) did not result in nodule formation. Lowercase letters in the figure show significant differences. Same letters mean non - significant group differences; different ones mean significant ones.

As shown in **Figure 5**, although nodules numbers and weights did not differ statistically between “R” and “R+GXU087” treatments, GXU087 inoculation increased nodule count from 128.8 to 148 and weight from 709.1 to 760 mg/plant. This indicates a synergistic effect of GXU087 and rhizobia on soybean nodulation.

### 3.5 Impact of extracellular extracts of GXU087 strain on soybean growth and nodulation

**Figure 6:**
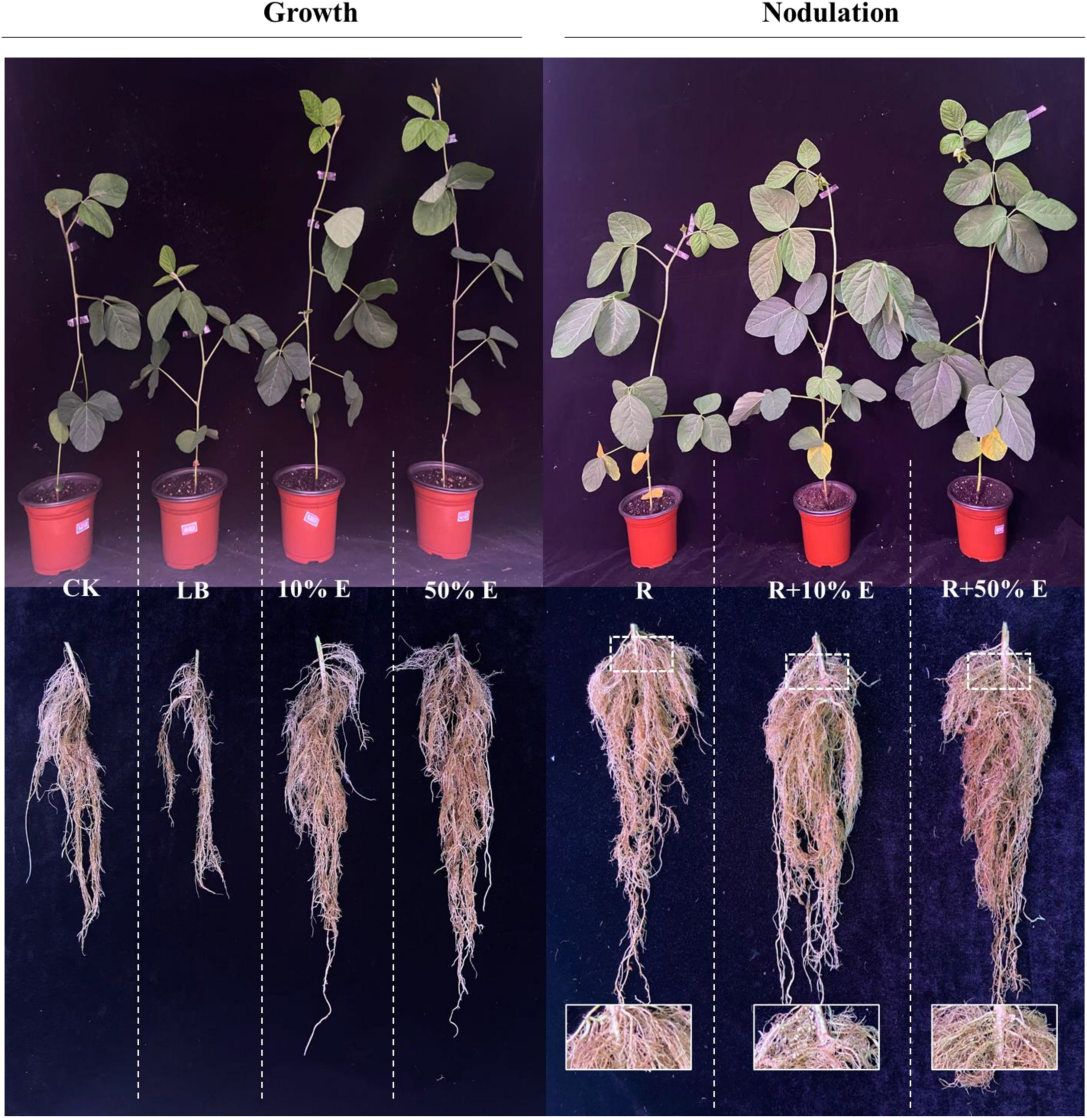
Overview of the growth and nodulation promotion by extracellular extracts of GXU087 strain in soybean. Growth assays (n=6) included treatments with SDW (CK), LB medium (LB), 10% extracellular extract (10% E), and 50% extracellular extract (50% E). Nodulation assays (n=10) included rhizobia inoculation alone (R), and with 10% extracellular extract (R+10% E) and 50% extracellular extract (R+50% E).

Many PGPB have been reported to promote crop growth by secreting growth-regulating substances, such as hormones. Therefore, we evaluated the impact of extracellular extracts of the GXU87 strain on soybean growth and nodulation in pot assays, and we observed a notable increase in shoot and root biomass with both the 10% and 50% of extracellular extracts treatment, as shown in **Figure 6**.

**Figure 7:**
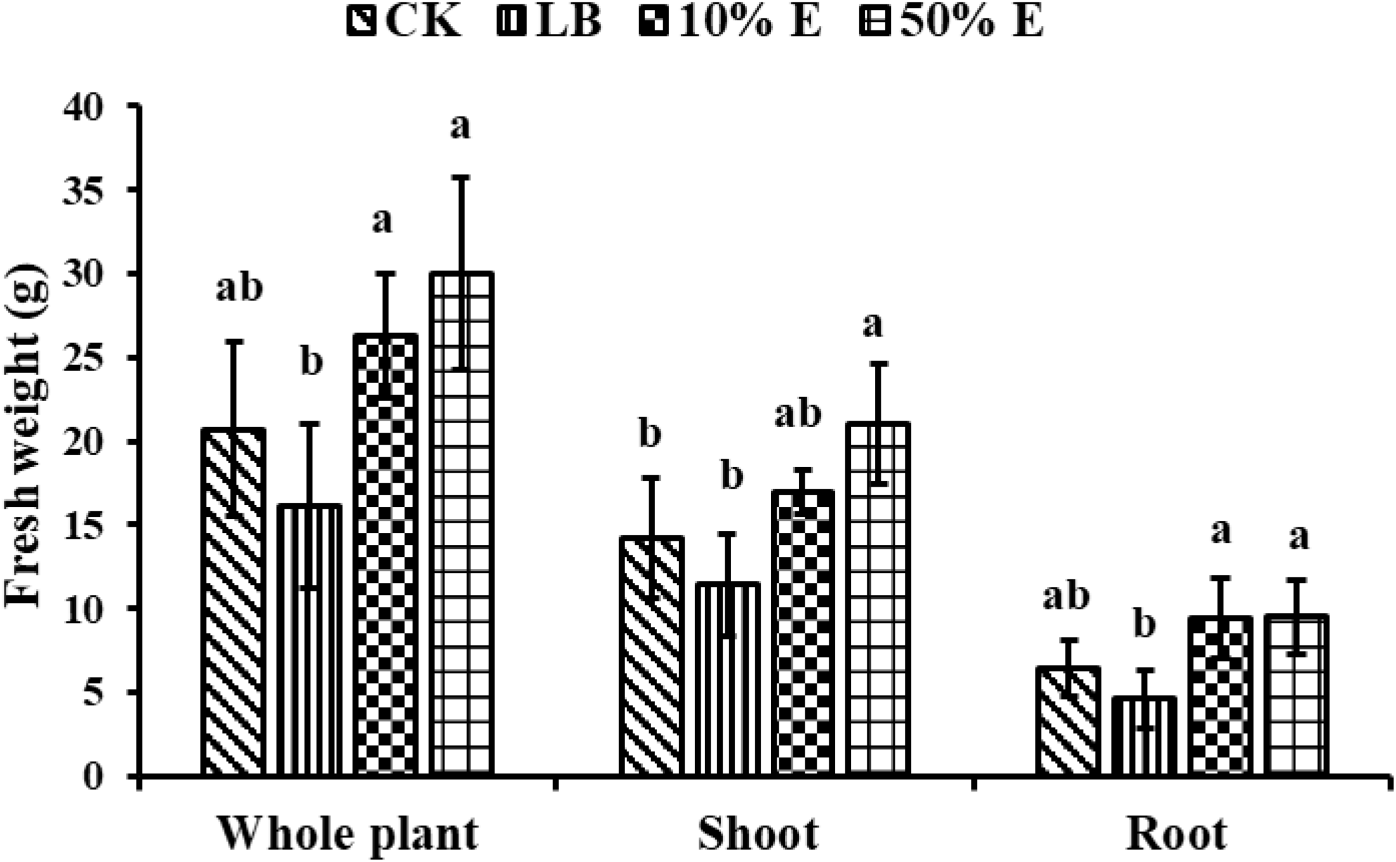
Growth promotion on the weight of soybean by extracellular extracts of GXU087 strain at 14 dpi. Significance at *p ≤0.05* is indicated by mean standard error bars (n=6). Growth assays included treatments with SDW (CK), LB medium (LB), 10% extracellular extract (10% E), and 50% extracellular extract (50% E). Lowercase letters in the figure show significant differences. Same letters mean non - significant group differences; different ones mean significant ones.

As shown in **Figure 7**, applying 10% extracellular extract (10% E) at 14 dpi significantly increased shoot fresh weight by 19.7% (from 14.2g to 17 g), root by 46.8% (from 6.4g to 9.4 g), and whole-plant fresh weight by 27% (from 20.7g to 26.3g), compared to the blank control. Applying 50% extracellular extract (50% E) significantly boosted fresh weight, with shoot weight by 47.8% (from 14.2g to 21g), root weight by 48.4% (from 6.4g to 9.5g), and whole-plant weight increasing by 44.9% (from 20.7g to 30g), compared to the blank control. These results indicate that extracellular extract of the GXU87 strain has a substantial stimulating effect on soybean growth.

**Figure 8:**
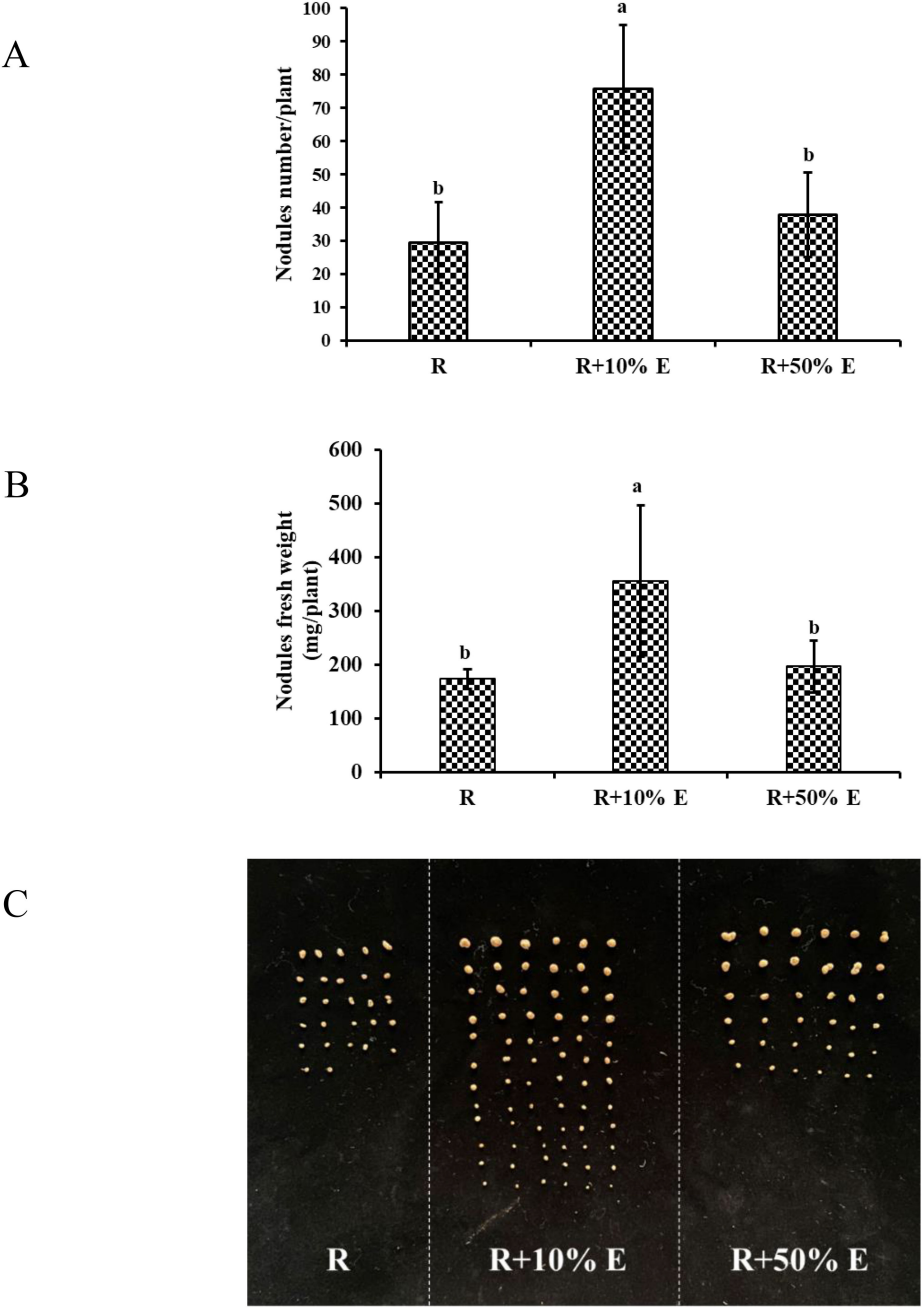
Nodulation parameter of soybean by irrigation with extracellular extracts of GXU087 strain at 21 dpi: **(A)** nodules number/plant; **(B)** nodules fresh weight; **(C)** photograph of soybean nodule. Significance at *p ≤0.05* is indicated by mean standard error bars (n=10). Nodulation assays included rhizobia inoculation alone (R), and with 10% extracellular extract (R+10% E) and 50% extracellular extract (R+50% E). Lowercase letters in the figure show significant differences. Same letters mean non - significant group differences; different ones mean significant ones.

As illustrated in **Figure 8**, applying 10% E significantly increased nodule count from 29.5 to 75.8 (*p* < 0.05) and the fresh weight from 173.8 mg/plant to 355.4 mg/plant (*p* < 0.05), whereas 50% E showed a non-significant increase in nodule count (29.5 to 37.8, *p* > 0.05) and fresh weight (173.8 mg/plant to 196.6 mg/plant, *p* > 0.05). This suggests that 10% E has a superior effect on nodulation.

### 3.6 UPLC-MS analysis of auxin profile reveals high levels of ILA in GXU087 extracts

**Figure 9:**
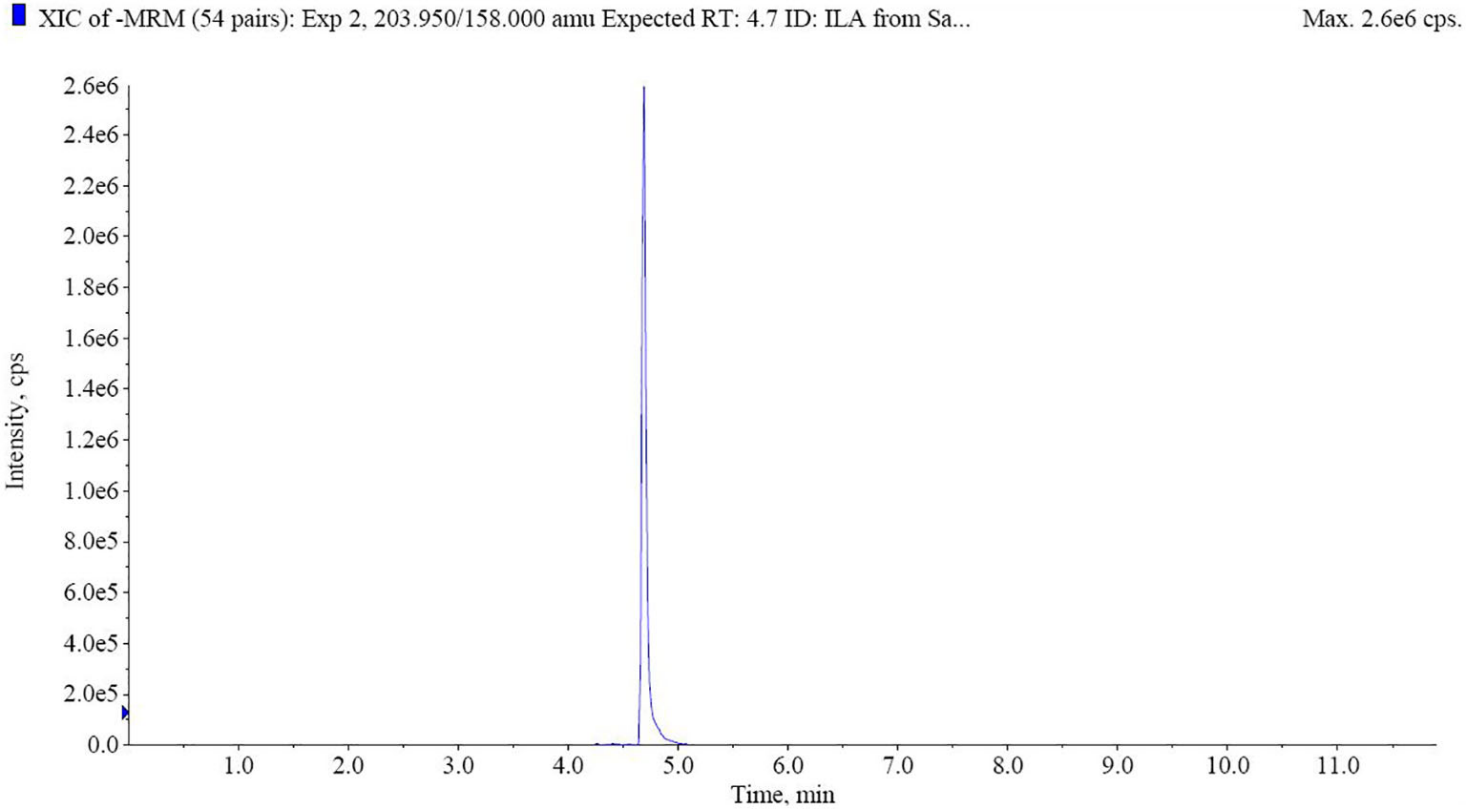
UHPLC-MS analysis of ILA from extracellular extracts. The retention time of ILA is 4.7 min.

UPLC-MS analysis revealed the abundant levels of ILA (232.7 ng/mL) in the exogenous extracts of GXU087. **Figure 9** demonstrates the identification of ILA in the extracellular extracts through UPLC-MS analysis.

### 3.7 ILA promotes soybean growth and nodulation

**Figure 10:**
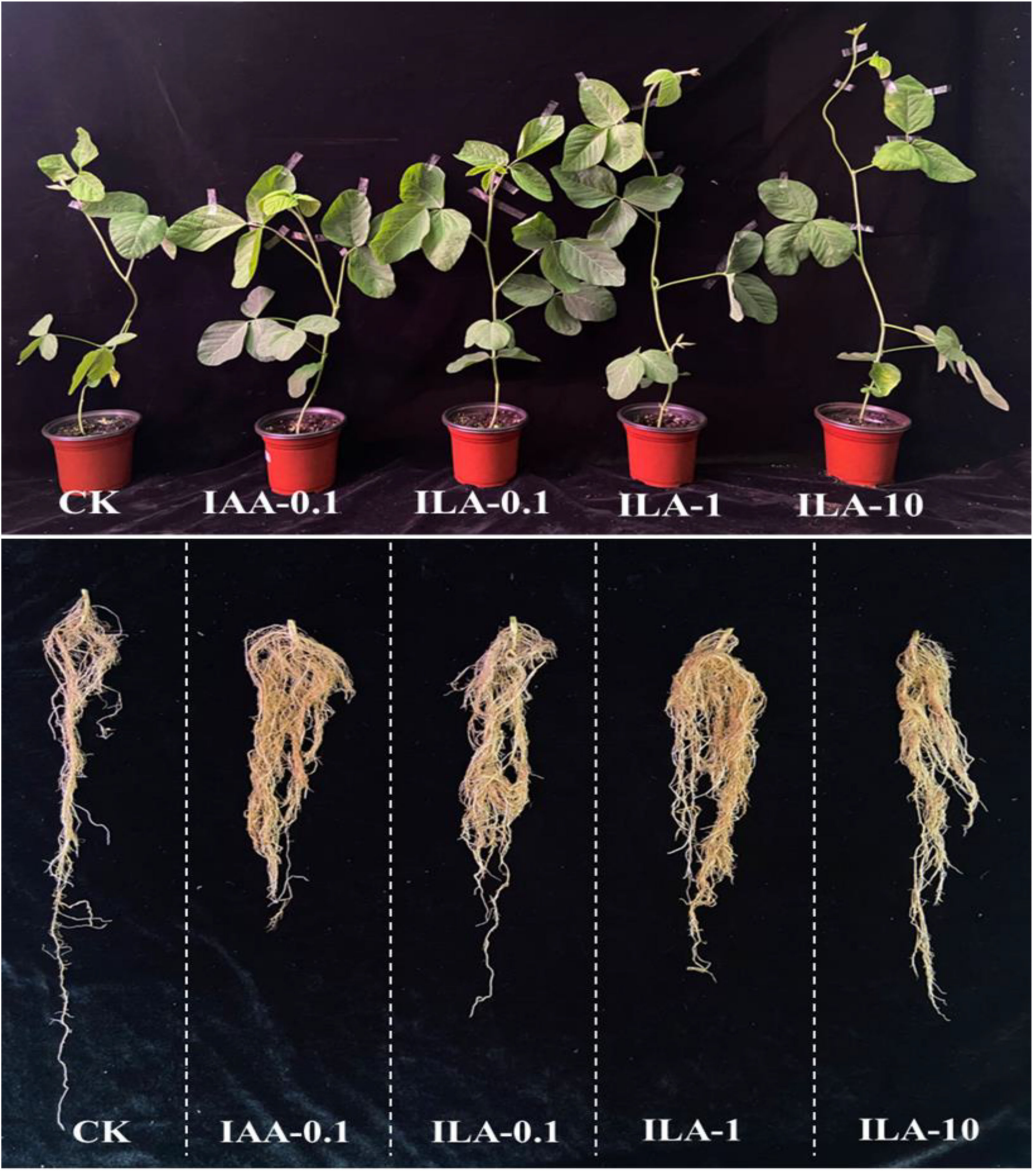
Overview of the growth promotion by irrigating ILA on soybean (n=6). CK: blank control group; IAA positive control group: IAA-0.1(irrigated with 0.1 mg/L of IAA); ILA-0.1: irrigated with 0.1 mg/L of ILA; ILA-1: irrigated with 1 mg/L of ILA; ILA-10: irrigated with 10 mg/L of ILA.

**Figure 11:**
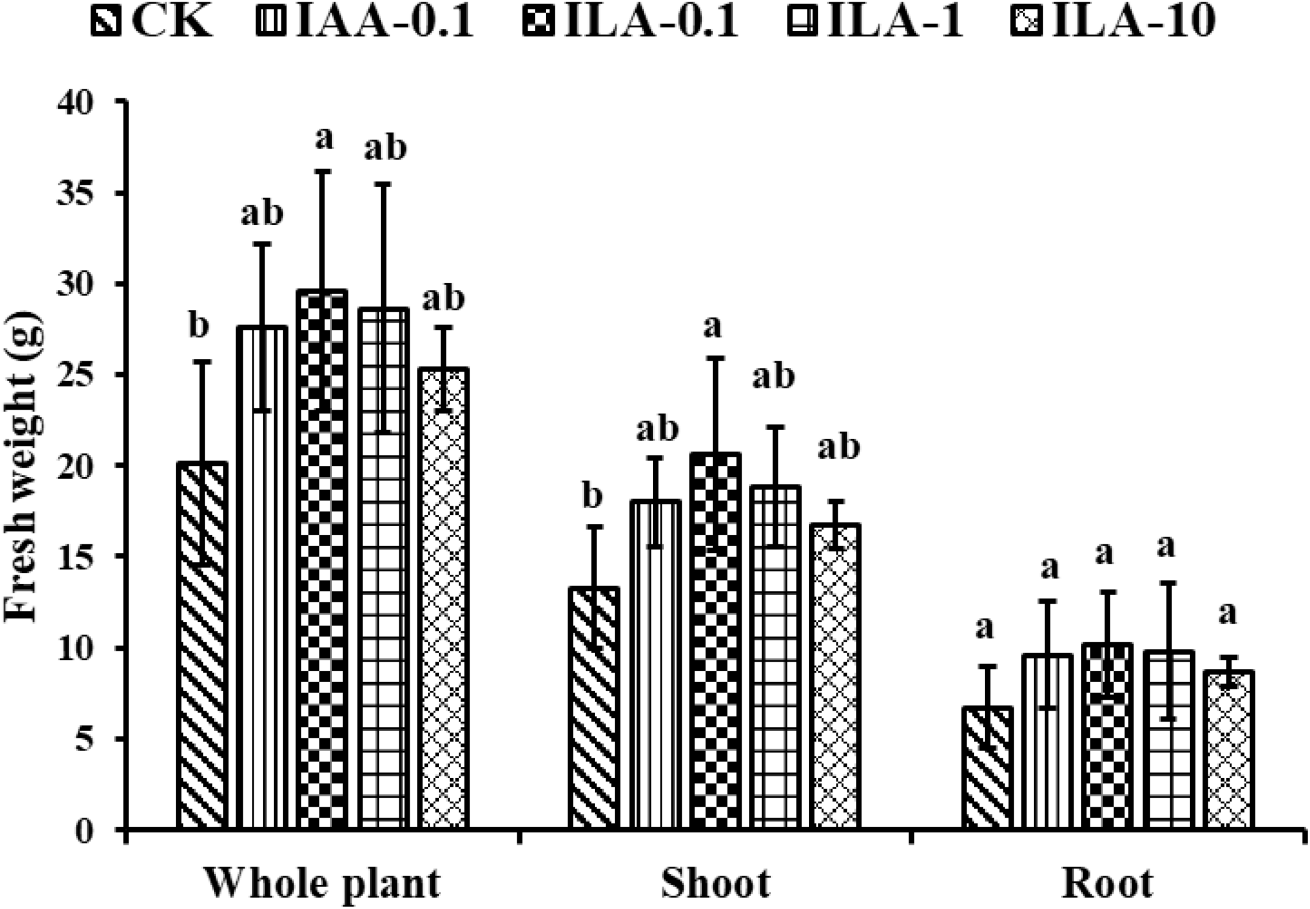
Growth promotion on the weight of soybean by irrigating exogeneous ILA at 14 days. CK: blank control group; IAA positive control group: IAA-0.1(irrigated with 0.1 mg/L of IAA); ILA-0.1: irrigated with 0.1 mg/L of ILA; ILA-1: irrigated with 1 mg/L of ILA; ILA-10: irrigated with 10 mg/L of ILA. Significance at *p* ≤0.05 is indicated by mean standard error bars (n=6). Lowercase letters in the figure show significant differences. Same letters mean non - significant group differences; different ones mean significant ones.

IAA is a crucial regulators of plant development, influencing elongation, proliferation, and differentiation through its auxin activities. The similarity of its molecular structure and abundance in extracellular extracts prompted us to explore the function of ILA. **Figure 10** demonstrated that ILA (0.1-10 mg/L) significantly enhances soybean shoot and root biomass. As shown in **Figure 11**, the lowest concentration (0.1mg/L) provided optimal growth stimulation compared to medium (1mg/L) and high (10mg/L) concentrations. At 0.1 mg/L, ILA increased whole-plant fresh weight from 20.1g to 29.6 g (*p* < 0.05), shoot fresh weight from 13.3 g to 20.6 g (*p* < 0.05), and root fresh weight from 6.7 g to 10.2 g (not statistically significant). Although the ILA exhibited a promotion in the soybean growth at the concentration of 1mg/L and10mg/L, it was not statistically significant. Additionally, ILA exhibited a comparable growth-promoting effect on soybeans as positive control (IAA), suggesting it is a weak auxin analogue for soybean.

**Figure 12:**
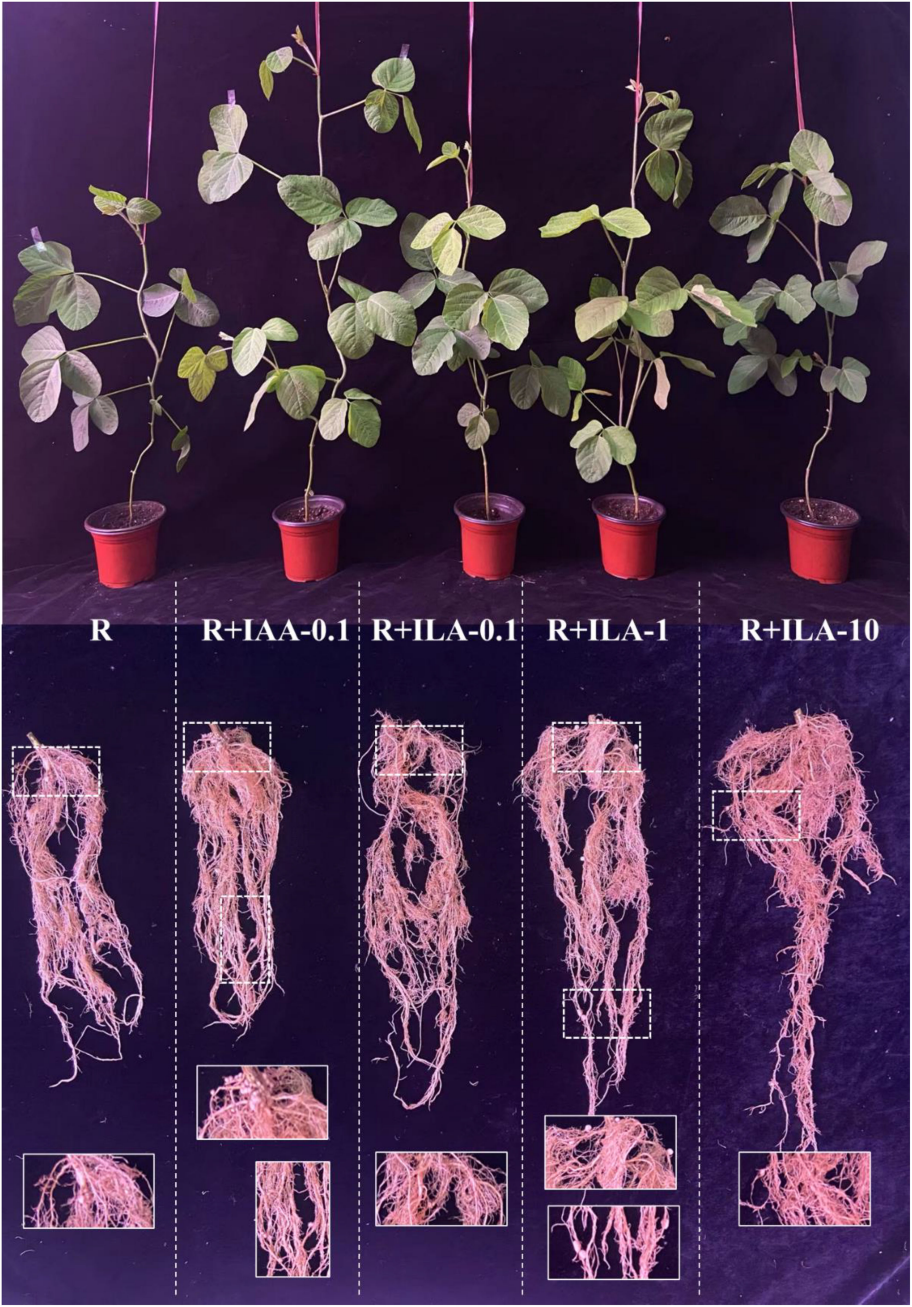
Overview of promotion of irrigating ILA on soybean growth in the present of rhizobia strain(n=10). R: rhizobia inoculation without exogeneous compound; R+IAA-0.1: rhizobia inoculation with 0.1 mg/L of IAA as the positive control; R+ILA-0.1: rhizobia inoculation with 0.1 mg/L of ILA; R+ILA-1: rhizobia inoculation with 1 mg/L of ILA; R+ILA-10: rhizobia inoculation with 10 mg/L of ILA.

**Figure 13:**
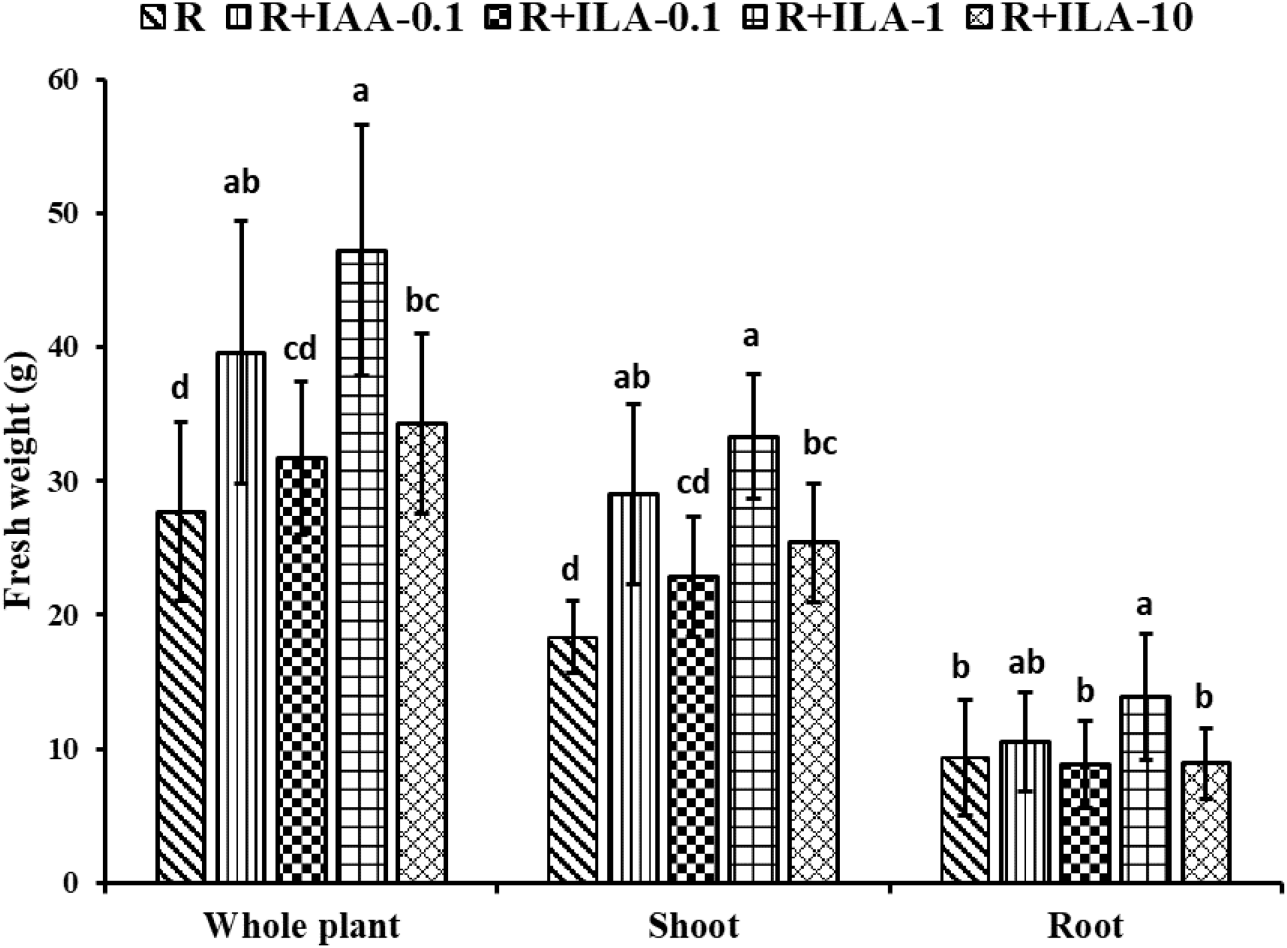
Growth promotion on the weight of soybean by irrigating exogeneous ILA at 21 days. R: rhizobia inoculation without exogeneous compound; R+IAA-0.1: rhizobia inoculation with 0.1 mg/L of IAA as the positive control; R+ILA-0.1: rhizobia inoculation with 0.1 mg/L of ILA; R+ILA-1: rhizobia inoculation with 1 mg/L of ILA; R+ILA-10: rhizobia inoculation with 10 mg/L of ILA. Significance at *p ≤0.05* is indicated by mean standard error bars (n=10). Lowercase letters in the figure show significant differences. Same letters mean non - significant group differences; different ones mean significant ones.

Interestingly, as seen in **Figure 12**, ILA applied in the presence of a rhizobia strain seemed to enhance the PGP effect of the strain. **Figure 13** demonstrated that co-inoculation with 1 mg/L of ILA and the rhizobia strain significantly increased whole-plant fresh weigh from 27.7 g to 47.2 g (*p* < 0.05), shoot fresh weight from 18.3 g to 33.3 g (*p* < 0.05), and root fresh weight from 9.3 g to 13.8 g (*p* < 0.05). This indicates a synergistic effect of IA and rhizobia on soybean growth.

**Figure 14:**
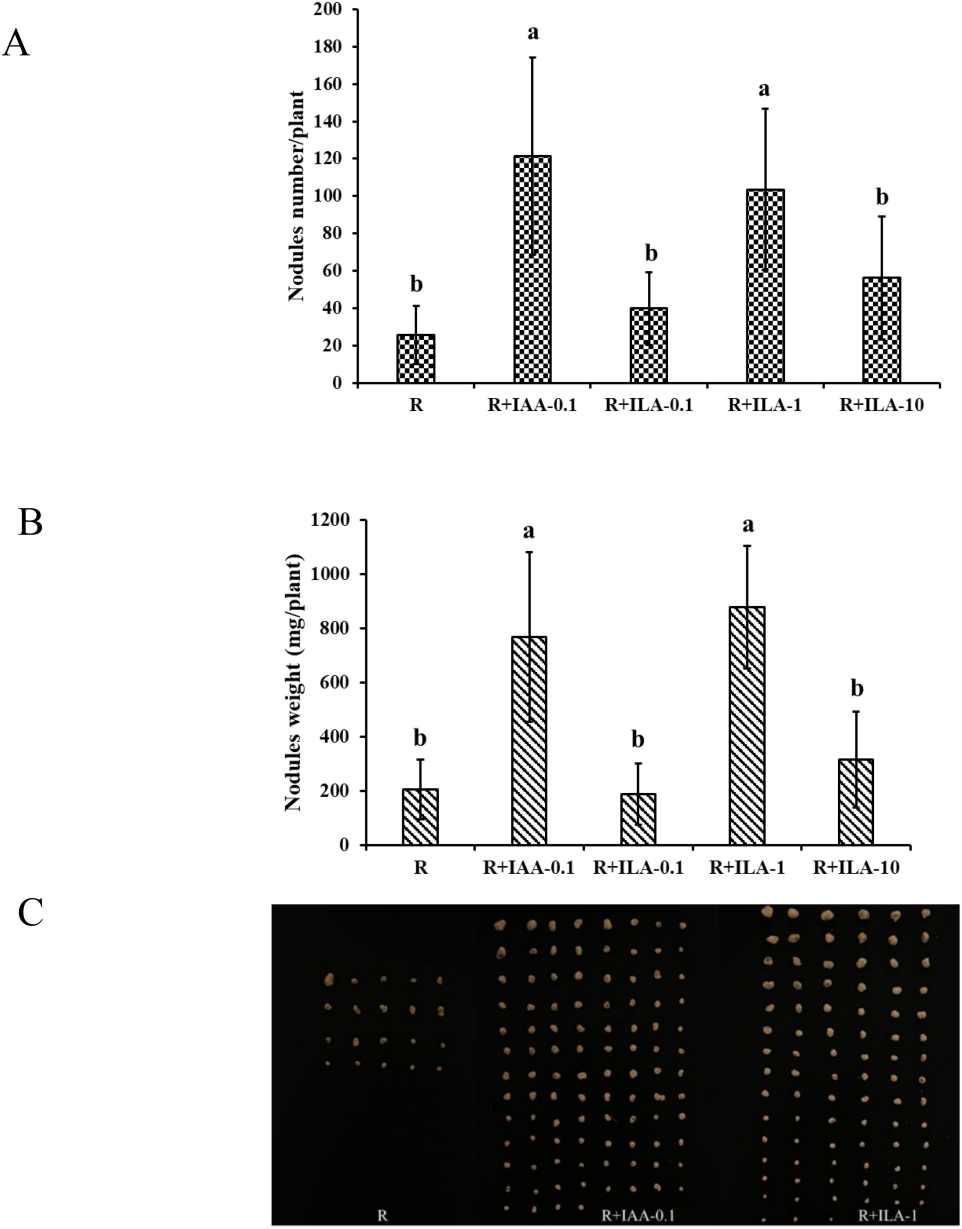
Nodulation parameter of soybean by irrigation with exogeneous ILA in the present of rhizobia strain at 21 dpi: **(A)** nodules number/plant; **(B)** nodules fresh weight; **(C)** photograph of soybean nodule. R: rhizobia inoculation without exogeneous compound; R+IAA-0.1: rhizobia inoculation with 0.1 mg/L of IAA as the positive control; R+ILA-0.1: rhizobia inoculation with 0.1 mg/L of ILA; R+ILA-1: rhizobia inoculation with 1 mg/L of ILA; R+ILA-10: rhizobia inoculation with 10 mg/L of ILA. Significance at *p* ≤0.05 is indicated by mean standard error bars (n=10). Lowercase letters in the figure show significant differences. Same letters mean non - significant group differences; different ones mean significant ones.

As shown in **Figure 14**, ILA significantly promoted nodulation at 1mg/L, increasing nodule count by 25.7 to 103.6 (*p* < 0.05), nodule fresh weight from 205 mg/plant to 878 mg/plant (*p* < 0.05). While ILA (1 mg/L) and positive control had similar effects on nodule number, ILA treatment resulted in larger and heavier nodules. In conclusion, this study demonstrates that ILA stimulates soybean growth and nodulation, performing similarly to IAA positive control.

### 3.9 ILA have no stimulation effect on the propagation of rhizobia population

The application of ILA did not significantly stimulate the growth dynamics of the rhizobia bacteria, as shown in **Supplementary Figure S8**. During the early phase (0-24 h), the growth of rhizobia that exposed to this substance was comparable to that of the control. After 24 h, ILA slightly inhibited rhizobia growth. This result indicates that the exogenous substances did not promote the reproduction of the rhizobia population. Therefore, we speculate that ILA’s promotion of soybean nodulation may be due to other mechanisms.

## Discussion

PGPB are microbes that promote plant growth and crop yield by enhancing phosphate solubilization, nitrogen fixation, and producing plant hormones such as auxins (Lima et al., 2024; Zahra et al., 2023; Ladha et al., 2016; Bhattacharjee and Dey, 2014). *Bacillus* sp. is an important PGPB genus in various crops, known for its ability to survive under harsh situations and to produce phytohormones (Gopalakrishnan et al., 2011; Zheng et al., 2019; Thomas et al., 2024; María et al, 2024) and exhibit high nitrogenase activity (Zahra et al., 2023). Numerous studies have shown that *Bacillus* sp. positively influences leguminous growth and nitrogen fixation (Kumar et al., 2021; Miljaković et al., 2022a; Sahile et al., 2021). In the current study, *B. megaterium* GXU087 significantly promoted the growth and nodulation of soybean. Our findings align with those of Miljaković et al. (2022b), who reported that *B. megaterium* significantly improved shoot length, root length, and seedling vigor index in cold-treated soybean seeds. Additionally, Kang et al. (2014) observed beneficial effects of *B. megaterium* mj1212 on the mustard plants. These studies suggested that *B. megaterium* is a promising bio-inoculant for soybean plants. However, despite extensive research on the PGP effects of *B. megaterium*, the mechanisms underlying these effects remain unclear. In this study, we screened a PGP strain, *B. megaterium* GXU087, from soybean fields. This strain exhibits multiple PGP traits, such as phosphate solubilization, nitrogen fixation, production of exopolysaccharide, and biofilm formation. This finding suggests that GXU087 is a promising PGPB strain. By enhancing nutrient solubilization and conferring resistance against plant pathogens, it holds great potential for achieving phytoremediation and promoting plant growth (Nur et al., 2023; Numan et al., 2018).

IAA was biosynthesized through multiple pathways in bacteria and play a crucial role in regulating plant growth and micro-microbe signaling (Spaepen et al., 2007). Many PGPBs attribute their PGP effects to IAA production (Sibponkrung et al., 2020; Jochum et al., 2019). For instance, Masciarelli et al. (2014) reported that *B. amyloliquefaciens* strain LL2012 produces high levels of auxin, gibberellins, and SA, promoting soybean growth and significantly improving nodulation in the presence of *Bradyrhizobium japonicum*. Sibponkrung et al. (2020) found that disrupting genes involved in IAA and CK biosynthesis in *B. velezenis* strain S141 resulted in smaller size nodules in soybean-*Bradyrhizobium* symbiosis, demonstrating the importance of hormones secreted by this strain for nodule growth.

In this study, extracellular extracts from GXU087 positively influence soybean growth and nodulation, indicating that GXU087 exerts PGP effects by synthesizing and secreting certain chemical substances. UPLC-MS analysis revealed high levels of ILA in the metabolites of GXU087. Subsequently, we found that exogenous ILA significantly improves soybean growth and nodulation as an auxin analogue. This is the first report of ILA acting as a promoter in soybean growth. Although ILA was previously considered a weak auxin analogue decades ago, Sprunck et al. (1995) reported that it was inactive for *Pisum sativum* L. at physiologic concentrations. This slight difference may be due to the difference of crop varieties and physiological state. In conclusion, B. megaterium GXU087 promotes soybean growth and nodulation through the production of ILA. This finding suggests a tryptophan-dependent pathways for IAA biosynthesis in GXU087 strain. However, the exact mechanism of ILA functions remains unclear. Gibson et al. (1972) reported that ILA might be converted into IAA in tomato shoots, explaining its weak auxin activity in tomato cotyledons. Previous studies have suggested that ILA acts as an IAA antagonist in plants by competing with IAA for binding to auxin carriers and transporters in vitro (Korber et al., 1991). However, newer findings contradict this hypothesis (Sprunck et al., 1995). It is unclear whether ILA serves as an intermediate in IAA biosynthesis in soybean. Consequently, further research is recommended to elucidate the underlying mechanism of bacteriogenic ILA in plant growth promotion.

In the past, the abundant of other metabolites secreted from *Bacillus* sp. were overlooked. Recently, certain indole derivatives, such as indole-3-pyruvic acid (IPA/IPyA) and indole-3-carboxaldehyde (IAAld), have been proven to be regulatory substance in PGP strain (Magdalena et al., 2012; Lu et al., 2024). Indole derivatives like IPyA, IAAld, indole-3-acetamide (IAM), indole-3-xethanol (TOL), indole-3-lactic acid (ILA), and tryptamine (TAM) serve as various intermediates in microbial IAA synthesis (Tang et al., 2023; Magdalena et al., 2012; Pan et al., 2023). Notably, indole-3-carboxaldehyde (ICAld) have been reported to be secreted by the PGPR strain Streptomyces lasalocidi, which alleviates salt stress in soybean (Lu et al., 2024). However, despite the discovery of numerous indole derivatives in microbial synthesis, their functions remain unclear.

## Conclusion

In this study, we isolated and identified a novel PGPB strain, *B. megaterium* GXU087, which exhibit various PGP traits. Notably, this strain promotes the soybean growth and nodulation through the secretion of exogeneous substance. UPLC-MS analysis confirmed that GXU087 secreted indole-3-lactic acid (ILA). Pot assays confirmed that ILA promoted soybean growth and nodulation. Consequently, we conclude that *B. megaterium* GXU087 promotes soybean growth and nodulation by secreting ILA. This is the first report of *B. megaterium* secreting ILA as a promoter of soybean growth. Despite the synthesis pathway of ILA by *B. megaterium* and the precise mechanism of ILA action remain unclear, our findings contribute novel perspectives on the underlying mechanisms of *B. megaterium*.

## Data Availability

The datasets presented in this study can be found in online repositories. The names of the repository/repositories and accession number(s) can be found in the article/**Supplementary Material.**
