# Supplementary material for: Bacillus megaterium GXU087 secretes indole - 3 - lactic acid to promote soybean growth and nodulation
Source: Front Plant Sci. 2025 Mar 21;16:1560346. doi: 10.3389/fpls.2025.1560346 (PMC11968722; doi:10.3389/fpls.2025.1560346)
Supplement: Supplementary file 1 [file DataSheet1.docx]

**Supplementary Material**


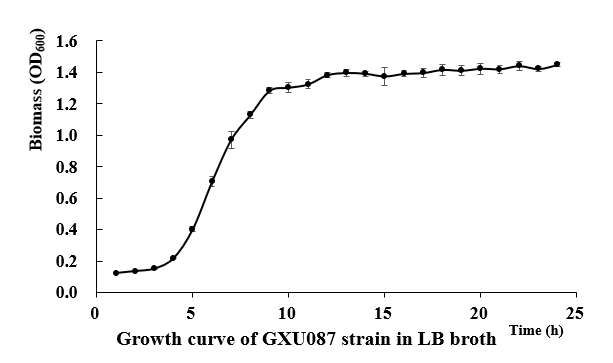


Figure S1. The growth dynamic of GXU087 strain in LB broth at 28 °C


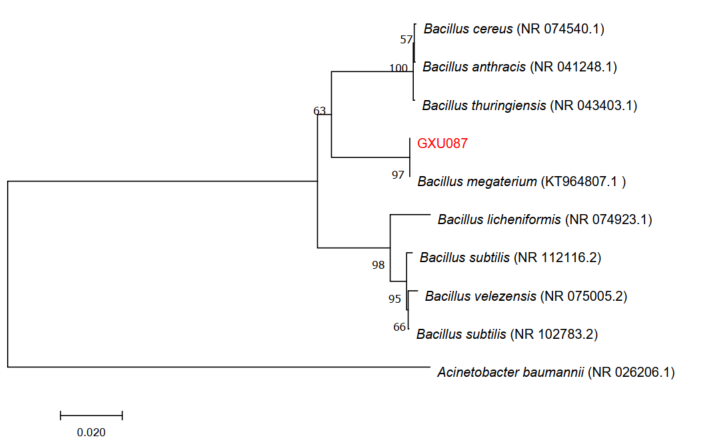


Figure S2. The phylogenetic tree of the GXU087 strain was constructed based on the 16S rRNA sequence. Bootstrap values are based on 1000 replications. Bar 0.02 substitutions per site.


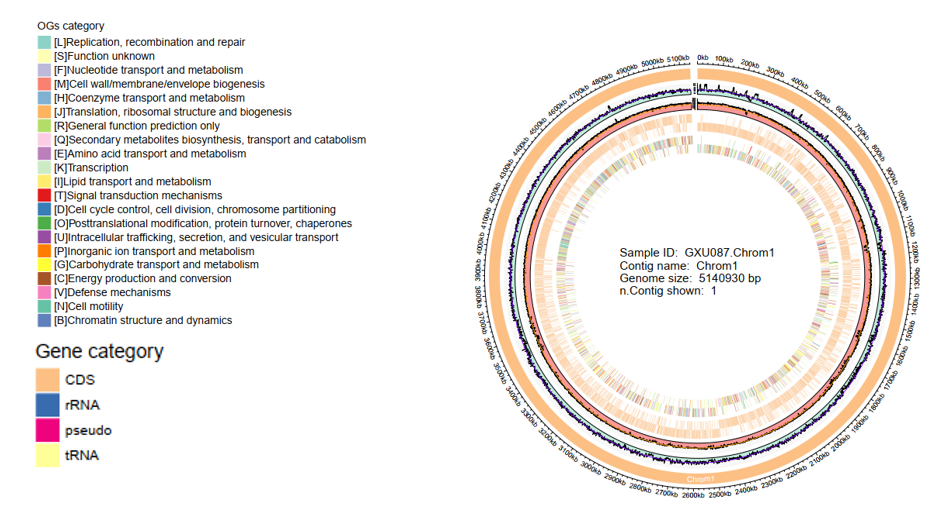


Figure S3. Circle map of the genome of *Priestia. megaterium* GXU087 chromosome. The genome sequences of the GXU087 strain have been deposited in NCBI under accession numbers CP144863.


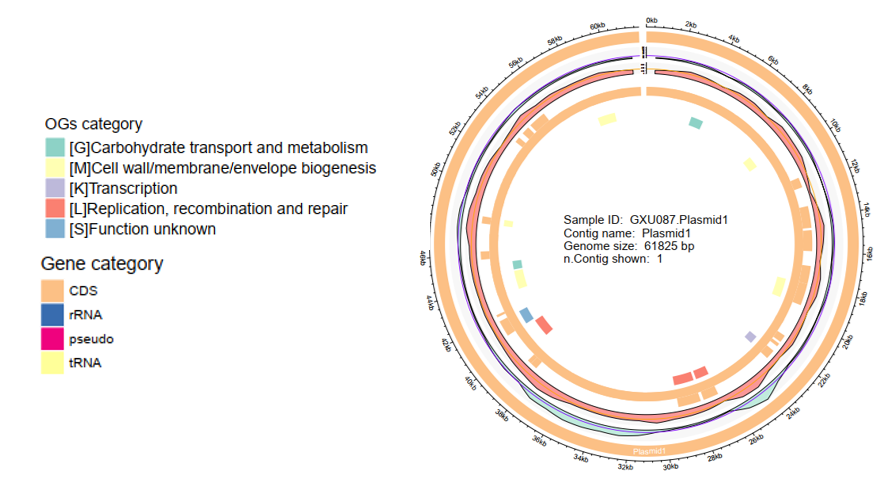


Figure S4. Circle map of the genome of *Priestia. megaterium* GXU087 plasmid 1. The genome sequences of the GXU087 strain have been deposited in NCBI under accession numbers CP144864.


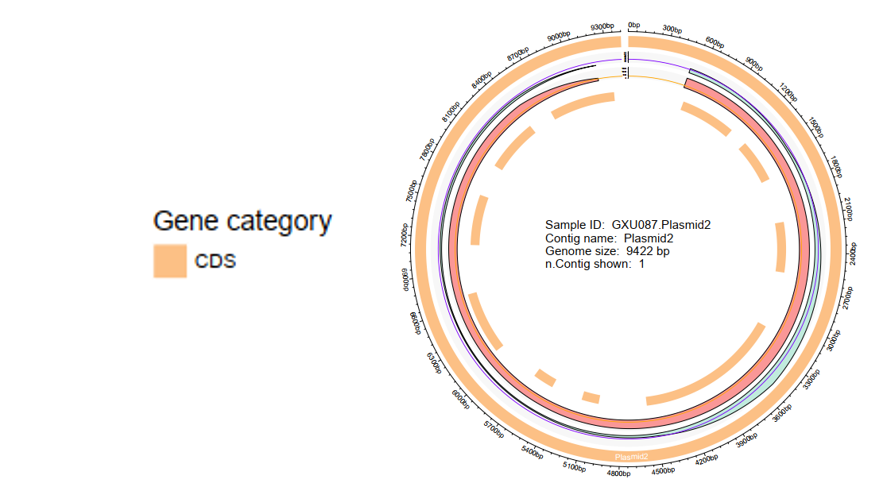


Figure S5. Circle map of the genome of *Priestia. megaterium* GXU087 plasmid 2. The genome sequences of the GXU087 strain have been deposited in NCBI under accession numbers CP144865.


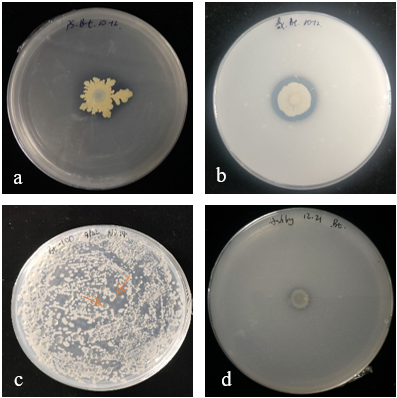


Figure S6. PGP traits assay. Clear zone formation around the colony in Pikovaskya's agar medium (Fig. S6a) and Mongina agar medium (Fig. S6b) indicated the phosphate solubilizing efficiency of GXU087. Thick slime (*mucoid*) formation (yellow arrows) around the colony in ATCC medium No.14 (Fig. S6c) indicated the exopolysaccharide (EPS) production. Clear zone formation around the colony in the Ashby plate indicated the nitrogen fixation capability of the GXU087 strain (Fig. S6d).


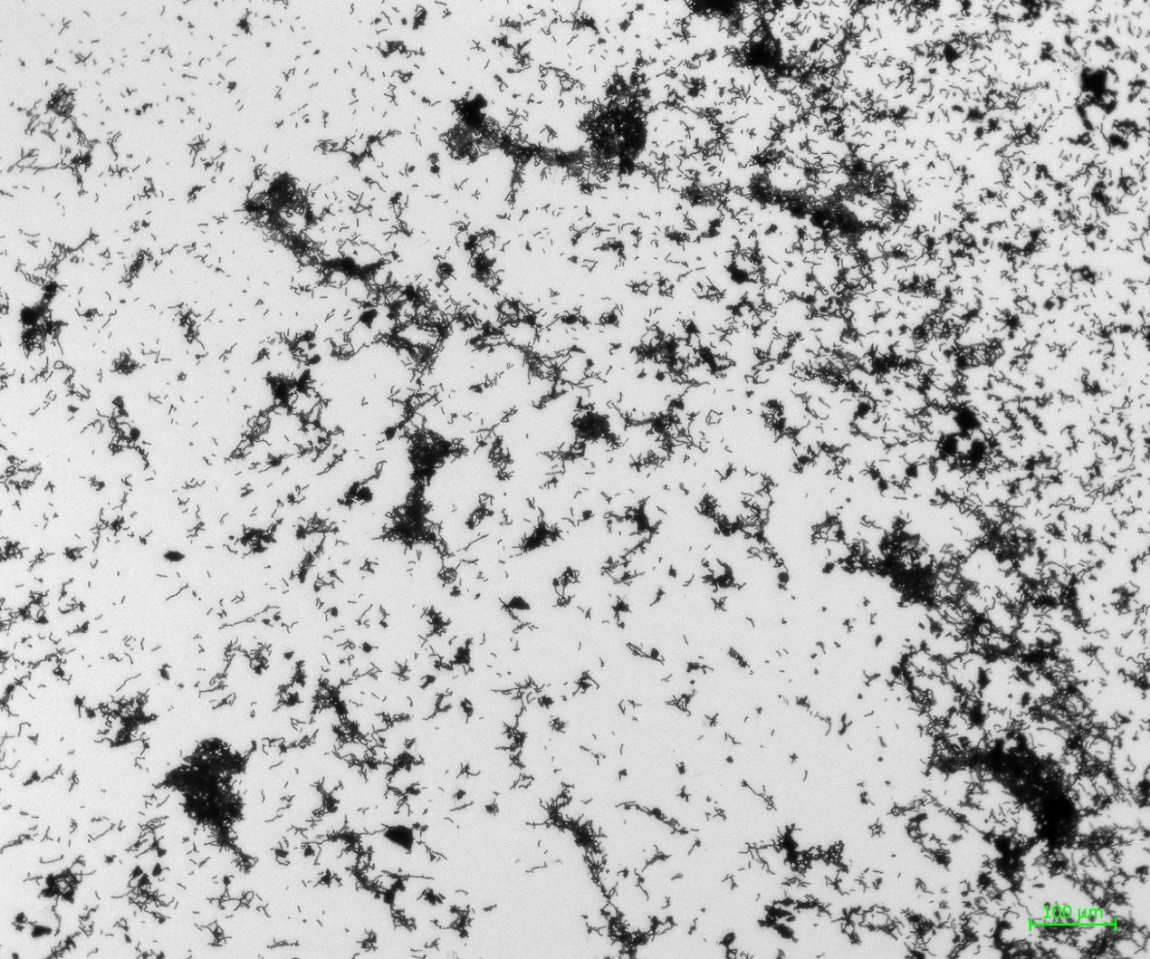


Figure S7. Biofilm formation of GXU087 stimulated by Msgg medium


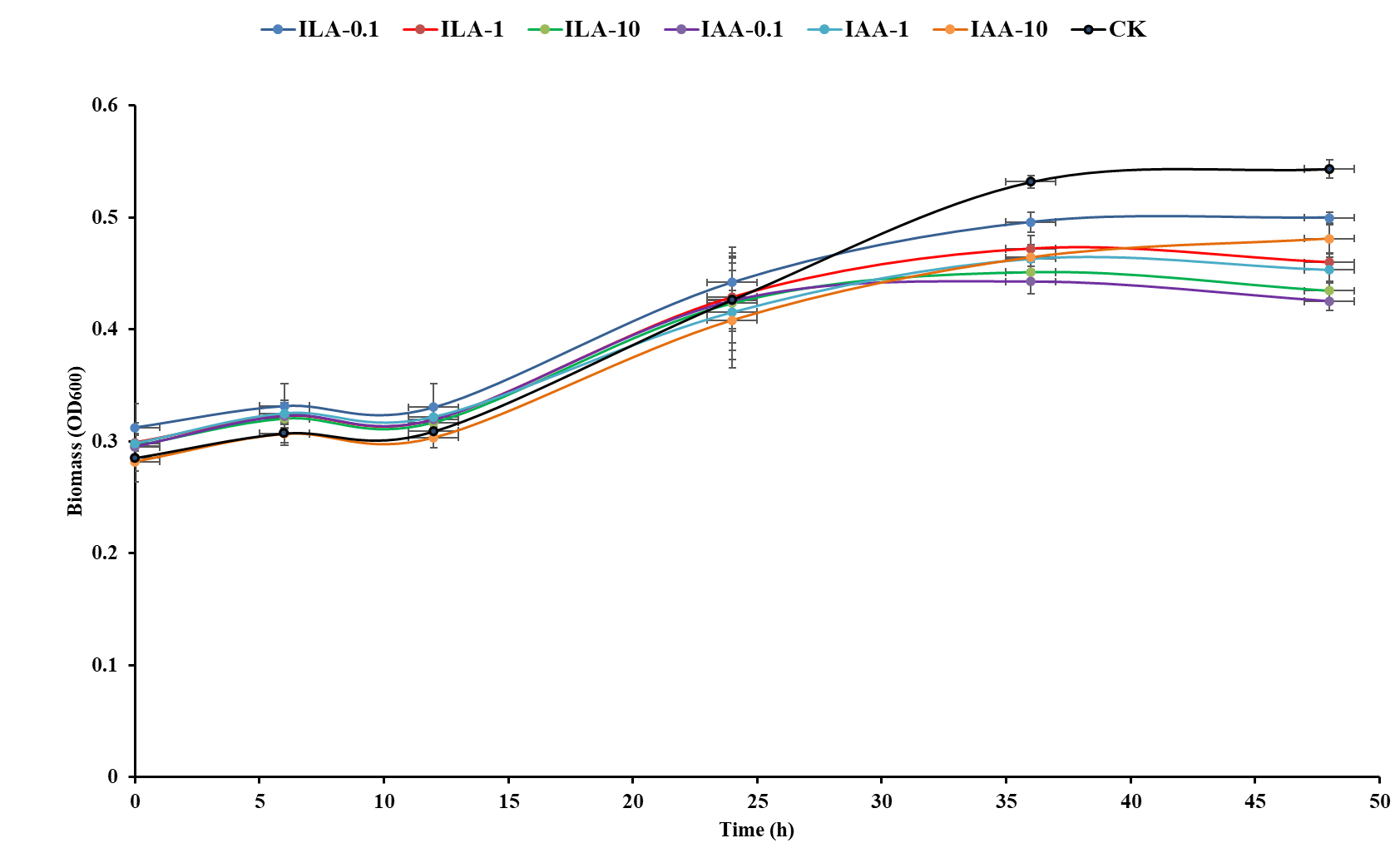


Figure S8. The influence of exogeneous substances on the growth dynamic of rhizobia strain.
